# Different components of air pollutants and neurological disorders

**DOI:** 10.3389/fpubh.2022.959921

**Published:** 2022-11-28

**Authors:** Chunlia Fu, Daibing Kuang, He Zhang, Jinxin Ren, Jialong Chen

**Affiliations:** ^1^Department of Emergency Intensive Care Unit, Affiliated Dongguan People's Hospital, Southern Medical University, Dongguan, China; ^2^School of Public Health, Guangdong Medical University, Dongguan, China; ^3^The Second Clinical Medical College, Guangdong Medical University, Dongguan, China

**Keywords:** air pollutants, different components, neurological disorders, nervous system, ambient air pollution

## Abstract

The harmful effects of air pollution can cause various diseases. Most research on the hazards of air pollution focuses on lung and cardiovascular diseases. In contrast, the impact of air pollution on neurological disorders is not widely recognized. Air pollution can cause various neurological conditions and diseases, such as neural inflammation, neurodegeneration, and cerebrovascular barrier disorder; however, the mechanisms underlying the neurological diseases induced by various components of air pollutants remain unclear. The present paper summarizes the effects of different components of air pollutants, including particulate matter, ozone, sulfur oxides, carbon oxides, nitrogen oxides, and heavy metals, on the nervous system and describes the impact of various air pollutants on neurological disorders, providing ideas for follow-up research.

## Introduction

Air pollution is a major global public health problem. Most people breathe clean air every day, whereas others live in areas with air pollution all their lives. Air quality is closely related to our quality of life, so the study of air pollutants is of great importance. According to the Global Burden of Disease report, air pollution is associated with the occurrence of many acute and chronic diseases. Air pollution was responsible for 6.5 million deaths worldwide in 2015. According to World Health Organization statistics of (1), ambient air pollution causes approximately 3.7 million premature deaths worldwide every year, 88% of which occur in middle-income and low-income countries. The exposure to a variety of pollutants in the air is the main reason for these deaths. Epidemiological results show that air pollutants cause central nervous system diseases and increase hospitalization rates and mortality rates (2). However, studies on the relationship between air pollution and the central nervous system are limited. Considering the complex effects of air pollution on the central nervous system, a large number of studies are needed to obtain more accurate data.

Many forms of pollutants are present in the air, including atmospheric particulate matter, ozone (O_3_), sulfur oxides, carbon oxides, nitrogen oxides (NO_x_), and heavy metals. These pollutants can directly or indirectly cause damage to human health, and their chemical composition changes with geographical location, seasonal changes, and alterations in the source; thus, the toxic effect also differs. A growing body of research suggests that living in areas with high levels of air pollution is associated with neurodegenerative diseases (3). Unlike other risk factors, such as excessive alcohol consumption, smoking, physical inactivity, and an unhealthy diet, it is difficult to make lifestyle choices to avoid or improve air pollution. Thus, improvements in the environment and air quality, especially polluting emissions, are not controlled by patients or doctors but by policy-makers who manage air pollution to protect citizens from adverse health effects. The first step to improving air quality is to review and consider environmental stressors, such as air pollution, as important hazards in official prevention guidelines (4). Therefore, it is particularly important to study various pollutants and diseases related to the nervous system.

In summary, this article will review recent studies that describe the influence of air pollutants, including atmospheric particulate matter and O_3_, sulfur oxide, carbon oxide, and nitrogen oxide, on the nervous system. We will describe various air pollutants and review the effects of air pollutants on common diseases of the nervous system.

## Search strategy

Seven English and Chinese electronic databases, including PubMed, Embase (Ovid), the Cochrane Library, SinoMed, China Science and Technology Journal Database (VIP), China National Knowledge Infrastructure (CNKI) database, and Wanfang Data Knowledge Service Platform, were searched from their inception to December 2021 by two reviewers for original research articles.

## Data extraction and quality assessment

All the search results were imported into the literature management software (Note Express V.3.4) for further screening. Two independent reviewers (MW, JR) conducted two rounds of literature screening. First, the reviewers read the title and abstract for preliminary screening, downloaded the full text of the papers that had passed the preliminary screening, and made a final judgment after reading the full text. After determining the literature that would be included in the final analysis, specific features of these articles were extracted using data extraction table.

### Damage caused by air pollutants and cerebrovascular disease

CVD is an important public health problem that is attracting increasing attention. In addition to risk factors, such as hypertension, diabetes, and hyperlipidemia, epidemiology suggests that PM_2.5_ correlates with **CVD**. PM_2.5_, or fine particles, have an aerodynamic diameter of 2.5 microns or less and are a major component of particles in the air. PM_2.5_ is the result of natural and human factors, mainly wood combustion, coal combustion, fuel combustion, and vehicle emissions. PM_2.5_ is small in volume, abundant in quantity, large on the surface, high in activity, and slow in sedimentation, and therefore, provides a medium for a variety of chemicals, bacteria, and viruses. Given its lightweight, it remains in the atmosphere for a long time and can be deposited in the alveoli through breathing (5). It can even penetrate the blood system and cause pathological changes.

A study on the relationship between PM_2.5_ and CVD found that for every 10 μg/m^3^ increase in PM_2.5_ concentration, the risk of emergency admission for CVD increased by 1.29%, indicating that PM_2.5_ concentration was positively correlated with the risk of emergency admission for CVD (6). Yang et al. evaluated the short-term effects of air pollution on stroke incidence and mortality and found that when PM_2.5_ increased by 10 μg/m^3^, the stroke hospitalization rate and fatality rate increased by 1.2%. These results indicate that PM_2.5_ is more likely to cause the occurrence of CVD and death in patients exposed to higher concentrations (7). A study by Hedges et al. involving 18,278 UK adults found an association between PM_2.5_ concentration and a smaller left hippocampal volume with a 10.78 mm^3^ reduction in left hippocampal volume for each unit increase in PM_2.5_ concentration (8). In other words, for each unit increase in PM_2.5_ concentration, the volume of the left hippocampus decreased by 0.28%. Other studies have found that the incidence and mortality of cardiovascular diseases in some countries or regions decrease with the decrease in PM_2.5_ concentration, and the life expectancy of residents is increasing (9), suggesting that PM_2.5_ exposure affects the cardiovascular health of residents. It was also found in animal experiments that exposure to PM_2.5_ could impair the number and function of vascular endothelial cells and prevent vascular recovery after limb ischemia mediated by endothelial cells (10).

### Air pollutants and Alzheimer's disease

#### PM_2.5_ and Alzheimer's disease

With the aging society, AD has become one of the main diseases affecting the health of elderly individuals. AD is a progressive neurodegenerative disease with cognitive dysfunction as the main clinical manifestation. A survey of people over the age of 65 years in Taiwan over the past decade found that PM_2.5_ in the atmosphere increased by 4.34 μg/m^3^ for every 10 years of the observation period, whereas the incidence of AD increased 138% (11). These findings indicate that the increase in PM_2.5_ concentration increases the risk of AD. A German study of older women with long-term exposure to traffic pollution found that traffic-related PM_2.5_ can cause cognitive decline (12). Air pollution components and cognitive function in the cognitively sound middle-aged and elderly population in Los Angeles found that compared with participants exposed to PM_2.5_ ≤ 15 μg/m^3^, people exposed to higher concentrations of PM_2.5_ have low language learning ability. These findings further demonstrate that high concentrations of PM_2.5_ are closely related to cognitive function (13). Similarly, animal experiments have found that female rats exposed to PM_2.5_ are more likely to show reduced spatial learning and memory accompanied by changes in the expression of inflammatory cytokines in the hippocampus and dendrite density and branching of hippocampal neurons (14). In addition, Bhatt et al. found that long-term inhalation of PM_2.5_ led to pathological changes similar to AD in the brains of transgenic mice (15).

#### Ozone and Alzheimer's disease

Stratospheric ozone is formed by the splitting of oxygen molecules by the sun's shortwave high-energy ultraviolet rays. Surface O_3_ is a secondary pollutant that is formed by the photochemical conversion of main pollutants, such as NO_x_ and volatile organic compounds, generated by human activities as precursors (16). O_3_ is a strong oxidizing pollutant that can cause nerve damage by inducing the release of free radicals, activating the production of inflammatory cytokines, and damaging the integrity of the blood–brain barrier. At present, an increasing number of epidemiological, clinical, and toxicological studies have shown that O_3_ is related to the occurrence and development of some neurodegenerative diseases.

A prospective cohort study conducted in the Taiwan Province of China found that with each increase in atmospheric O_3_ concentration, people aged 65 years and older had a 2.11-fold increased risk of AD (17). Lo et al. investigated the effects of long-term exposure to ambient air pollution on the cognitive function of 2,241 elderly people living in the community and found that long-term exposure to O_3_ was significantly correlated with cognitive impairment [odds ratio (OR) = 1.878, 95% confidence interval (CI): 1.363–2.560] (18). Cerza et al. found a strong association between long-term O_3_ exposure and hospitalization for AD with a 20% increase in the risk of hospitalization for every 10 μg/m^3^ increase in summer O_3_ (19). In animal models, O_3_ exposure has also been found to induce increased interleukin (IL)-17 expression, primarily in hippocampal neurons, along with chronic neurodegenerative changes associated with activation of hippocampal astrocytes similar to those observed in human AD (20). Studies have also found that long-term exposure to low doses of O_3_ can lead to oxidative stress and irreversible progressive neurodegeneration (21). Another animal study found that long-term exposure to low levels of O_3_ can cause oxidative stress and damage striatal and substantia nigra neurons (22). Although most studies have shown the harmful effects of O_3_ on cognition, other studies have shown a protective effect of O_3_ on cognitive function. A combination of air pollution in the daily detailed data on 1,054 participants from all over Taiwan was used to explore the relationship between air pollutants and cognitive impairment. The results show that exposure to different air pollutants can result in cognitive decline in the general and specific areas of cognitive impairment, and O_3_ may serve as a protective factor (23). A high concentration of O_3_ correlated with a high cognitive total score, suggesting that O_3_ may have a neuroprotective effect on cognitive function.

#### Sulfur dioxide and Alzheimer's disease

SO_2_ is a widespread air pollutant that is mainly emitted from sulfur-containing fuels, such as those used in power plants. Although the literature on its potential neurological effects remains limited, some existing studies suggest that chronic ambient air pollution exposure may have an impact on neurological disorders.

Lin et al. used case–control and urban control studies to compare the progress of AD patients in cities with different levels of air pollution. In this study, clinical data on 704 AD patients from 2002 to 2018 were retrospectively collected, and it was found that high SO_2_ exposure had the greatest effect on cognitive deterioration in AD (HR = 1.24, 95% CI: 1.14–1.35, *P* < 0.001) (24). Chen et al. also found a negative correlation between SO_2_ and overall cognitive decline and impairment in specific functional areas (including orientation, recall, and language), indicating that SO_2_ has a negative impact on neurocognitive performance (23). It has been found in animal experiments that SO_2_ exposure leads to synaptic dysfunction in the hippocampus, which may be related to the occurrence of cognitive impairment (25). Based on the effects of SO_2_ at different concentrations and exposure times in rats, researchers found that inhalation of SO_2_ can cause synaptic damage, which may be caused by protein kinase A (PKA) and/or protein kinase C (PKC)-mediated signaling pathways (26). The combination of PM_2.5_, SO_2_, and nitrogen dioxide (NO_2_) can impair spatial learning and memory and lead to abnormal expression of apoptosis-related genes (P53, Bax, and Bcl-2) (27). Another study found that the combined effect of SO_2_ and PM_2.5_ led to the accumulation of Aβ in the cerebral cortex and hippocampus of rats (28). However, other studies have found that SO_2_ may alleviate the injury caused by chronic cerebral hypoperfusion by enhancing the antioxidant capacity of the hippocampus (29). In addition, it has been reported that low doses of PM_2.5_ and SO_2_ coexposure can lead to neurodegenerative changes, including neuronal apoptosis and reductions in synaptic structural and functional proteins (30). SO_2_ is an air pollutant associated with the trajectory of impaired cognitive function, and it is necessary to further study its potential damage to cognition.

#### Carbon monoxide and Alzheimer's disease

CO, which is tasteless, colorless, and insoluble in water, accounts for a large proportion of air pollutants. According to statistics, the world emits ~26 Mt of CO into the atmosphere every year, 60% of which is derived from human activities. Anthropogenic CO emissions are mainly produced by incomplete combustion of carbon materials, most of which are emitted by gasoline engine vehicles. Other common emission sources include waste incineration and coal-fired power generation.

CO has 200 times the affinity for hemoglobin than oxygen, and a reduction in oxygen's binding ability leads to a reduction in the amount of oxygen reaching surrounding tissues, including the brain. Acute CO exposure can cause tissue hypoxic ischemia, heart damage, and shock, which may further aggravate hypoxia of the central nervous system, leading to nervous system damage (31, 32).

Nakamura et al. reported that chronic CO exposure led to elevated serum carboxyhemoglobin levels in two elderly patients with cognitive impairment. On their initial visit, both patients had cognitive impairment and brain magnetic resonance imaging findings consistent with the diagnosis of AD. However, when patients stopped using their charcoal heaters, their serum carboxyhemoglobin levels returned to normal, their physical symptoms disappeared, and their cognitive function improved slightly, suggesting that CO exposure may have temporarily exacerbated the patients' cognitive impairment (33). Chang et al. used data from the National Health Insurance Research Database on 29,547 people, including 1,720 dementia patients between 2000 and 2010, and assessed the risk of dementia with air pollution levels. The results of this large, population-based retrospective study suggest that exposure to CO is associated with an increased risk of dementia in the Taiwanese population. The negative effects of CO on cognitive performance were confirmed (34). Shin et al. also reported the associations between CO exposure and poor overall cognitive ability, poor attention, and poor executive function (35). Chen also found that high CO exposure was correlated with lower Mini-Mental State Examination (MMSE) total scores and recall domain scores, indicating the adverse effect of CO on cognitive function (23). In addition, Chen et al. confirmed that chronic CO poisoning could lead to microstructural damage and cognitive impairment in the corpus callosum by diffusion tensor imaging (36). Previous studies have also shown that smoking is highly associated with an increased risk of AD (37). Smoking exposes individuals to the toxic ingredients in tobacco smoke, such as CO. Interestingly, although smoking is associated with vascular dementia, several studies have reported adverse effects of smoking on the neurobiology and function of the brain in people without a history of cardiovascular disease (38). This finding may indicate that CO plays a role in cognitive decline.

#### Nitrogen dioxide and Alzheimer's disease

According to statistics, human industrial production and social activities emit 52 million tons of NO_x_ into the atmosphere every year. As the main form of NO_x_, NO_2_ is an important target of air pollution and environmental quality. NO_2_ enters through the respiratory tract, corrodes and irritates the respiratory tract, and causes respiratory tract damage. After entering the body, NO_2_ first targets the lungs. When the lungs exchange gas, NO_2_ enters the blood and then reaches the tissues and organs of the organism through the circulation of body fluids. Brain tissue is the most important part of the central nervous system of the organism. The brain is also the hub of the nervous system. The learning, memory, and cognitive functions of the organism are closely related to brain function. Therefore, brain damage caused by NO_2_ and its transformed derivatives in living organisms are of great concern.

Chen et al. conducted a cohort study of 2.1 million people to assess whether air pollution exposure is associated with dementia. In this study, a high level of NO_2_ was associated with an increased incidence of dementia from 2001 to 2013 (HR = 1.10; 95% CI: 1.08–1.12) (39). Crous-Bou et al. investigated the effects of urban environmental exposure on cognitive performance and brain structure in healthy individuals at risk for AD found that higher NO_2_ exposure was associated with lower cortical thickness in the hippocampus (40). Li et al. showed that NO_2_ was a potential risk factor for vascular dementia, and the synaptic (trigger) mechanisms that induce neuronal excitation in normal and stroke rat models were elucidated (41). Molton and Yang summarized the relationship among air pollution, oxidative stress, and AD and provided evidence that exposure to air pollutants may lead to chronic oxidative stress, which is the main pathogenesis of AD (42). Chang et al. examined the relationship between exposure levels of atmospheric NO_2_, CO, and AD. The study was based on 29,547 residents from the National Health Insurance Research Database in Taiwan, China, including 1,720 patients diagnosed with dementia between 2000 and 2010. This study estimated the risk of dementia at four levels of air pollution (quartiles of NO_2_ or CO concentration) and correlated detailed daily air pollution data from January 1, 1998, to December 31, 2010. We calculated the date from baseline to the onset of dementia, patient withdrawal, or study end along with the average annual pollutant concentration. We segmented or classified the distribution of these data by quartiles and concluded that exposure to high levels of NO_2_ or CO increases the risk of AD in Taiwan, China (34). In animal models, NO_2_ inhalation in mice was also found to cause a decrease in spatial learning and memory, increase amyloid β42 (Aβ42) accumulation, and promote pathological abnormalities and cognitive deficits associated with AD (43). NO_2_ may exacerbate the ultrastructural impairment of synapses, neuronal damage, and synaptic plasticity (41).

#### Nitrogen oxides and Alzheimer's disease

Nitrogen oxide compounds exclusively consist of nitrogen and oxygen and include a variety of compounds. Most of the anthropogenic nitrogen oxide emissions are derived from the combustion of fossil fuels, such as automobiles, airplanes, internal combustion engines, and industrial furnaces. Nitrogen oxide is also derived from the process of producing and using nitric acid, such as nitrogen fertilizer in plants, organic intermediate plants, and non-ferrous and ferrous metal smelters. There are some reports indicating that NO_x_ affects the human body through respiration.

Studies have linked NO_x_ exposure to the development of AD. Cerza et al. recruited 350,844 subjects aged 65–100 years without dementia and followed them for 12 years to assess the association between long-term exposure to air pollution and first-time hospitalization for dementia. NO_x_ exposure was positively associated with hospitalization for dementia (NO_x_: HR = 1.01; 95% CI: 1.00–1.02/20 μg/m^3^) (19). Oudin et al. studied 1,806 subjects recruited in northern Sweden. The subjects were followed to study the long-term exposure to traffic-related air pollution and to assess the relationship between the incidence of dementia from 15 years since Birch's longitudinal study of dementia incidence data. The study found that subjects in the highest exposure group were more likely to be diagnosed with dementia than those in the lowest exposure group (HR = 1.43, 95% CI: 0.998–2.05), suggesting that NO_x_ levels may be an important risk factor for dementia development (44). Grande's recent population-based longitudinal study, which explored the relationship between long-term exposure to air pollution and dementia, examined and followed 2,927 participants over the age of 60 years who did not develop dementia. The study found that for every 8.35 μg/m^3^ increase in the average NO_x_ concentration in the dwelling over a 5-year follow-up period, the risk of dementia increased by 1.14-fold [1.14 (95% CI, 1.01–1.29)]. NO_x_, a common marker of traffic-related air pollution, has also been linked to higher rates of AD in longitudinal studies in Sweden and China (34, 44).

#### Manganese and Alzheimer's disease

Manganese is a trace metal commonly found in the environment and a component of urban air pollution with concentrations varying based on geography, season, and source (45). The main sources of manganese in the air include industrial activity and automobile exhaust. Industrial activity mainly involves iron and gold production, iron and steel manufacturing, and coke oven emissions. When manganese compounds are used as antiknock agents in automobile exhaust mainly generated by gasoline, atmospheric levels of manganese may also increase (46). Manganese is transported to the central nervous system either as free ions or as a non-specific protein (47).

Exposure to manganese is typically higher in an occupational manganese environment compared with a normal environment. People with occupational exposure have been reported to have deficits in attention and concentration, memory, visuospatial function, language, executive function, and other cognitive functions, and a dose–response relationship is noted between blood manganese levels and cognitive function (48). After exposure to manganese, women with higher levels of manganese in their blood had lower visual memory scores, whereas men with higher levels of manganese in their blood had lower initial learning and/or recall scores on visual and verbal tests (49). In two rural Mexican communities living in manganese mining areas, higher blood manganese levels were associated with lower cognitive function in the MMSE (50). Additionally, at a mining site in Mexico, airborne manganese levels were linked to attention impairment in adults (51). In a ferromanganese alloy factory in Brazil, manganese in mothers' hair, as measured by Raven's Progressive Matrices, was negatively correlated with non-verbal cognition (52). In two communities near a ferromanganese smelting plant in Brazil, manganese levels in hair and nails were inversely associated with visual working memory and intelligence (53). In a cross-sectional study, adults living in Marietta and East Liverpool, Ohio, USA, were exposed to high levels of industrial airborne manganese, and manganese exposure was associated with lower working memory, visuospatial memory, and language skills (54). Tong et al. showed that whole-blood manganese levels were inversely correlated with scores on various cognitive and neuropsychological tests with individuals with lower manganese levels scoring higher on mini-mental state tests (55). Interestingly, they also observed an increase in plasma Aβ peptide with increased manganese levels, suggesting that whole-blood manganese levels may be involved in the progressive cognitive impairment of AD. After exposure to manganese, the expression of APLP1 protein in apoptotic neurons and glial cells increased, leading to the formation of Aβ plaques in the frontal cortex, which resulted in cognitive and working memory impairment in animals (56). Together, these studies suggest that exposure to high levels of manganese leads to cognitive decline in adults.

#### How air pollution may trigger Alzheimer's disease

The mechanisms underlying the effects of air pollutant inhalation on the brain remain poorly understood, but evidence suggests that inflammation and oxidative stress are common features of disease processes caused by air pollutants (3). Animal studies have found that components of air pollution can invade the brain and lead to the upregulation of inflammatory cytokines, potentially causing central nervous system disorders (57–59). Microglial activation associated with neuroinflammation has been identified as a major factor in AD pathogenesis (60). Microglia, a type of glial cell, are the first and most important line of immune defense in the central nervous system. Microglia continuously eliminate necrotic nerve plaques and infectious substances in the central nervous system (61). However, upon conditions of overstimulation, microglial activation can lead to an increase in tumor necrosis factor (TNF)-α, IL-1β, interferon (IFN)-γ, and reactive oxygen species (ROS) production, which when left uncontrolled can be neurotoxic (62). Spangenberg and Green found that activated microglial cells play an important role in the pathogenesis of AD (60). Glial cells exposed to PM_2.5_ undergo morphological changes, suggesting that microglia may participate in PM-induced neuronal damage (63). Greve et al. demonstrated that O_3_ exposure impaired the ability of microglia, the brain's parenchymal immune cells, to associate with and form a protective barrier around Aβ plaques, leading to augmented dystrophic neurites and increased Aβ plaque load (64). We hypothesize that air pollution promotes an early AD-like phenotype dependent on neuroinflammation, especially activated microglia, which may play a key mediating role in neuroinflammation.

### Damage caused by air pollutants and Parkinson's disease

#### PM_2.5_ and Parkinson's disease

PD is the second most common neurodegenerative disease with insidious onset and unknown etiology. Currently, it is believed that PD may be related to aging, family genetics, and the environment. A study found that the effects of short-term exposure to PM_2.5_ on hospitalizations and all-cause deaths from PD, and other diseases increased by an average of 10 μg/m^3^ over a 2-day period, the PD hospitalization rate increased by 3.23%, and all-cause mortality increased by 0.64% (65). From 1999 to 2010, a study in 50 southeastern cities of the United States followed a population of 10 million elderly individuals, analyzed PD cases, and found that people exposed to PM_2.5_ for a long time increased their PM_2.5_ concentration by 1 μg/m^3^ per year. The risk ratio of hospitalization for PD was 1.08 (95% CI: 1.04–1.12) (66). These studies suggested that regardless of short-term or long-term exposure to PM_2.5_, the PD hospitalization rate was correlated with PM_2.5_. In addition, Levesque et al. found that the level of midbrain alpha-synuclein increased in mice after exposure to PM_2.5_ at a concentration of 992 μg/m^3^, indicating that PM_2.5_ can cause pathological changes in early PD (67).

#### Ozone and Parkinson's disease

In recent years, an increasing number of relevant publications have shown that long-term exposure to ambient air pollution can significantly increase the risk of PD, and O_3_ is one of the risk factors. Based on the 2001 Canadian Census Health and Environment Cohort, including a total of 3.5 million people, scientists found that long-term exposure to O_3_ is positively correlated with PD mortality. For each unit increase in O_3_ (10.1 PPB), the risk of PD mortality increased 1.09-fold (95% confidence interval 1.04–1.14) (68). Hu et al. conducted a meta-analysis to assess the association between long-term exposure to ambient air pollution and PD and found that long-term exposure to O_3_ increased the risk of PD, and each 1 PPB increase in O_3_ exposure increased the risk of PD by 1.01 times (OR = 1.01, 95% CI: 1.00–1.02) (69). Kirrane et al. examined the association between air pollution and PD in a rural population in a case–control study and found a positive association between O_3_ and PD risk in some regions during the warm season (April to November) (OR = 1.39; 95% CI: 0.98–1.98), but no significant association was reported between year-round O_3_ exposure and PD risk (70). In animal studies, O_3_ exposure also led to significant reductions in motor activity, lipid peroxidation, morphological changes, fiber loss, and dopaminergic neuron cell death in mice (21, 71). A decrease in the number of dopamine neurons may represent one of the causes of PD. Pereyra-Munoz et al. found that O_3_ exposure led to a significant decrease in motor activity. O_3_ induces lipid peroxidation, morphological changes, fiber loss, and cell death of dopaminergic neurons. The expression of dopamine- and cAMP-regulated neuronal phosphoprotein-32 (DARPP-32), inducible nitric oxide synthase (iNOS), and superoxide dismutase (SOD) correlated with O_3_ after repeated exposure. These changes indicate that O_3_ induced oxidative damage to the substantia nigra and striatum in rats (22). In addition, the experimental results showed that repeated exposure to low doses of O_3_ produces a state of chronic oxidative stress in biological systems and causes progressive dopaminergic neuron death in the substantia nigra in the absence of other pathological factors. The loss of redox balance leads to the increased oxidative metabolism of dopamine, which leads to increased levels of oxidized dopamine, leading to a vicious cycle that accelerates the progression of PD (DAQ) (71).

#### Nitrogen dioxide and Parkinson's disease

A matched case–control study in Denmark found that for every quartile increase in NO_2_ (2.97 MCG/m^3^), the risk of PD increased by 9% (72). Yuchi et al. collected a health database of 45- to 84-year-old residents (*N* ~ 678,000) in downtown Vancouver, Canada, and investigated the relationship between air pollution exposure and PD. They found that air pollutants were associated with the incidence of PD, and every quartile increased the NO_2_ concentration with a PD hazard ratio of 1.12 (1.05–1.20) (73). It has been reported that PD is not random but spatially dependent. A positive correlation between NO_2_ concentration and the incidence of PD has been reported (74). Although there have been some reports of an association between NO_2_ pollution and PD, studies of exposure to NO_2_ pollution to date have yielded inconsistent results. Liu et al.'s study assessed the impact of air pollution on PD risk. Limited evidence of an association between exposure to environmental NO_2_ and the risk of PD was found in an embedded case–control analysis that included 1,556 self-reported doctor-diagnosed Parkinson's cases between 1995 and 2006 and 3,313 controls matched for age, sex, and race with patients (75). A recent matched case–control study in the Netherlands also reported no significant association between PD and long-term exposure to outdoor NO_2_ (76).

#### Carbon monoxide and Parkinson's disease

Reports on the effects of CO on PD are limited, and early reports suggest that survivors of CO poisoning may exhibit complex syndromes, including Parkinson's characteristics or behavioral changes (77). It has also been reported that CO-associated PD is characterized by symmetrical limb stiffness, bradykinesia, gait disturbance, and postural instability (78–81). In a population-based cohort study, Lee et al. found that short-term CO exposure was significantly associated with PD exacerbation [1.46 (1.05–2.04) per 0.1 PPM] (82). Hu et al. conducted a systematic review and meta-analysis to assess the association between long-term exposure to ambient air pollution and PD with a combined risk of 1.65 (95% CI: 1.10–2.48) associated with increased CO exposure per part per million (83). Despite the limited number of reports on the effect of CO on PD, the available reports show a strong correlation between CO and PD. It is expected that more studies will explore the effect mechanism of CO on PD.

### NOx and Parkinson's disease

Several reports indicate that NOx exposure can increase the risk of PD. Li et al. reviewed 11,117 patients with PD from the Taiwan National Health Insurance Research Database and examined the effect of NOx in air pollution on PD in a longitudinal age- and sex-matched population of 44,468. A positive correlation was found between NOx exposure and PD with ORs of 1.37 (95% CI = 1.23–1.52) for PD in NOx above the 75th percentile compared to the lowest percentile in the multipollutant model. This finding suggests that exposure to ambient air pollution, particularly traffic-related pollutants, such as NOx, increases the risk of PD (84). A case–control study by Ritz et al. assessed the association between long-term traffic-related exposure to air pollutants and PD in 1,696 hospital-registered patients with PD and 1,800 controls matched by sex and year of birth. The study found a positive correlation between traffic-related pollutants (NO_2_, NOx) and PD. For each 7.1 μg/m^3^ increase in NOx concentration, the risk of PD increased by 1.06 (95% CI = 1.02–1.11) (72). In a systematic review and meta-analysis on the relationship between ambient air pollution and PD, Hu et al. found a significantly increased risk of PD with 10 parts per billion increase in NOx (RR = 1.06; 95% CI: 1.04–1.09) (69).

#### Manganese and Parkinson's disease

Excessive accumulation of manganese in the central nervous system can cause neurotoxicity, leading to a disease called manganese intoxication, which is characterized by early psychotic symptoms and often accompanied by chronic symptoms similar to idiopathic PD (85). This condition was first described by Couper (1837) in workers exposed to excessive manganese in the air. Parkinsonian manganese poisoning is known to result from long-term exposure to high levels of manganese in welding, smelting, and mining industries; battery manufacturing; ferroalloy mining; and manganese-rich agricultural chemicals (86). In the early stages of the disease, patients with manganese poisoning exhibit psychotic symptoms that progress to chronic disorders of extrapyramidal circuits, resulting in postural instability, dystonia and bradykinesia, microstrabismus, mask-like facial expressions, and speech disorders (87, 88). The toxic effects of manganese may be related to the imbalance between the dopaminergic and cholinergic systems, mainly in the striatum of the basal ganglia (89). Manganese causes the death of dopaminergic neurons through direct neurotoxicity and activates microglia to release inflammatory factors that accelerate neuron death.

Several epidemiological studies have assessed the association between manganese exposure and PD. In a cohort study of 886 US workers, a dose-dependent relationship was noted between exposure to manganese-containing welding fumes and the progression of PD (90). Similarly, in manganese miners in South Africa, cumulative manganese exposure is associated with Parkinson's signs and poorer quality of life (91). Abuse of mecazenone, a psychostimulant that uses potassium permanganate as an oxidant, has also been associated with extrapyramidal syndromes (92). However, other authors have not reported cognitive deficits, and neuropathological studies of methcathinone users have not been performed to elucidate the association between methcathinone users and the development of PD.

#### The mechanisms by which air pollution triggers Parkinson's disease

**Figure 1:**
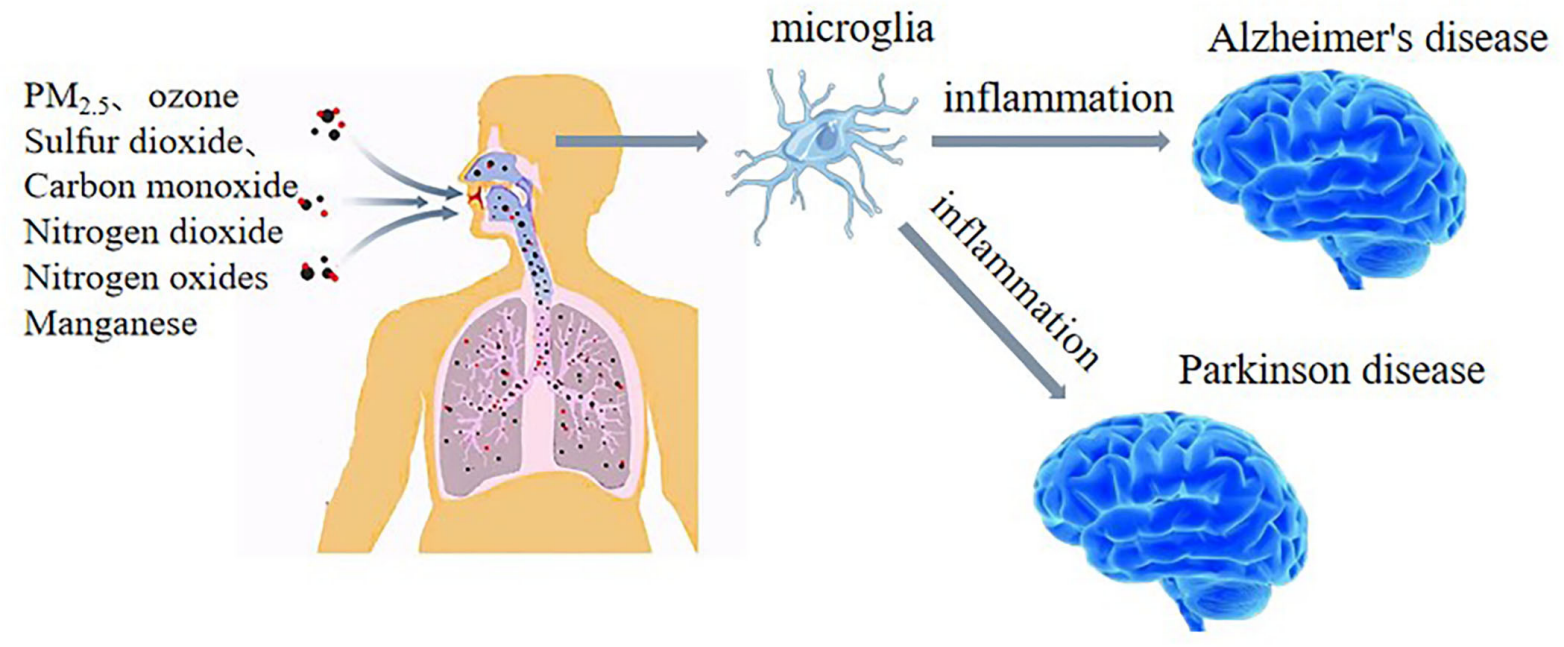
Air pollution increases neuroinflammation, especially microglial activation, which may be a key mechanism involved in air pollution-induced AD and PD.

In the past few years, there has been increasing concern regarding whether air pollution may be a risk factor for PD. Growing evidence suggests that neuroinflammation is a causal factor in PD, and exposure to air pollution is associated with brain inflammation and oxidative stress (93, 94). These processes are thought to contribute to the development and progression of PD (95). Autopsy, *in vivo*, and epidemiological studies support the role of inflammation in the pathogenesis of PD (95). Studies have shown that microglia play an important role in inflammatory mechanisms. Air pollution activates microglia in the human body, which may lead to the development and aggravation of alpha-synucleinopathy, a major component of the pathogenesis and development of PD (96). A study of children and young adults who died suddenly in Mexico City found that those residing in highly polluted urban areas showed increased neuroinflammation indicated by the presence of activated microglia and the accumulation of proteins, including alpha-synuclein and amyloid β42 (Aβ42) (97), compared with those residing in less polluted areas. These findings suggest the mechanism by which air pollution may promote the development of PD (see Figure 1).

### Role of air pollutants in multiple sclerosis

#### PM_2.5_ and multiple sclerosis

MS, a common neurodegenerative disease affecting ~2.5 million people worldwide, is caused by inflammatory demyelination of the brain, often resulting from autoimmunity causing progressive impairment of function (motor, sensory, visual, and cognitive) (98). Several studies have shown that environmental PM_2.5_ exposure increases the risk of MS. Farahmandfard et al.'s systematic review took the key words of air pollution as the exposure quantity, searched for “multiple sclerosis” as a result, and finally included 17 studies after applying inclusion and exclusion criteria. The results showed that outdoor air pollution, especially PM_2.5_, may be related to the prevalence or recurrence of MS (99). In a population-based cross-sectional study, Tato et al. found that the prevalence of MS was higher in urban areas vs. rural areas in Italy and that the prevalence was strongly correlated with the annual mean concentration of PM_2.5_, suggesting that PM_2.5_ may represent a possible environmental risk factor for MS (100). Elgabsi et al. examined the association between the incidence of moderate to severe MS recurrence and air pollutant and meteorological exposures and found that PM was independently associated with MS recurrence in nonsmokers [relative risk (RR) = 1.28, PV = 0.037] (101). Further studies found that the prevalence of MS in the population was increased >2-fold in highly polluted areas compared with less polluted areas (102). However, it has also been reported that air pollution is not associated with the risk of MS. Palacios et al. performed a large study that examined the risk of particulate exposure and MS found that exposure to PM air pollution was not associated with MS risk (103). Bai et al. did not observe a significant correlation between adult MS incidence and long-term exposure to PM_2.5_ in a large population-based cohort study (104). At present, there is still very limited evidence of a link between air pollution and MS incidence, and more population surveys are needed to assess the correlation.

#### O3 and multiple sclerosis

Epidemiological evidence supporting the role of O_3_ in the pathogenesis of MS is limited. Jeanjean et al. investigated the short-term association between O_3_ exposure and the recurrence rate of MS in 424 patients living in the Strasbourg region of France between 2000 and 2009 (a total of 1,783 relapsing cases) and found a significant correlation between the recurrence rate of MS and O_3_ exposure. Interestingly, these correlations are seasonal. Using the multipollutant model, only O_3_ was significantly associated with the probability of relapse during the “hot” season (105). Based on the Canada 2001 census queue of health and the environment, Zhao et al. found that mortality and MS increased as the O_3_ quartile range increased, and the associated fully adjusted HR for MS mortality was 1.35 (1.20–1.51) (68). Moghadam et al. recently explored the impact of air pollution on the prevalence, morbidity, mortality, and disease burden of MS worldwide. Using statistical methods of correlation analysis and linear regression analysis, the study showed that the prevalence, morbidity, and mortality of MS were inversely related to O_3_ concentration (β = −1.04, −0.04, and −0.01, respectively; *P* < 0.01) (106). A meta-analysis of the relationship between ambient air pollution and MS by Tang et al. showed that O_3_ was not associated with MS (69). Given the inconsistent results of previous studies, further research is needed.

#### Sulfur dioxide and multiple sclerosis

Studies on MS and SO_2_ are limited. Heydarpour et al. analyzed the spatial distribution of MS epidemics and their relationship to spatial patterns of air pollution. A regression model was used to estimate long-term exposure to SO_2_ in MS patients, and the results showed a clustered pattern of MS epidemics. The study revealed the potential role of long-term exposure to air pollutants as an environmental risk factor for MS (107). A case–control study of MS in children by Lavery et al. found that SO_2_ was associated with increased rates of MS in children (108).

#### CO and multiple sclerosis

A case–control study of MS in children by Lavery et al. found that CO was associated with increased rates of MS in children within 20 miles of an MS center (108). The results of the relationship between ambient air pollution and MS showed a variety of differences. Tang's study clarified and quantified the relationship between ambient air pollution and MS through meta-analysis, and the results showed that CO was not associated with MS (69). Thus, evidence in support of an association between CO and MS is limited and inconsistent.

#### Nitrogen dioxide and multiple sclerosis

Heydarpour et al. analyzed the spatial distribution of MS prevalence and its relationship with spatial patterns of air pollution. By collecting records of 2,188 patients who met the criteria for a clear diagnosis of MS from 2003 to 2013, a regression model was used to estimate the long-term exposure of MS patients to NO_2_. The results showed a cluster of MS epidemic cases in Tehran, and the spatial distribution of NO_2_ exposure was significantly different from that of MS epidemic cases, revealing the potential role of long-term exposure to NO_2_ as an environmental risk factor for MS (107). Jeanjean et al. used a case-crossover design to investigate the short-term association between NO_2_ exposure and the risk of MS recurrence in 424 patients with MS living in the Strasbourg region of France between 2000 and 2009 (1,783 relapsing cases). These correlations are seasonal. For example, a one-quarter-fold increase in NO_2_ exposure was associated with the incidence of MS recurrence (OR = 1.08; 95% CI: 1.03–1.14) in the “cold” season (October–March) (105). However, varied results on the relationship between NO_2_ and MS have been reported. In a population-based cohort study that examined the relationship between MS incidence and long-term exposure to NO_2_, Bai et al. did not observe a significant association between MS incidence and long-term exposure to NO_2_ during 13 years of follow-up (104). Tang et al. further clarified and quantified the relationship between ambient air pollutants and MS through meta-analysis and found that NO_2_ was not associated with MS (69).

#### Nitrogen oxides and multiple sclerosis

Current evidence for NO_x_ and MS is limited. Heydarpour et al. analyzed the spatial distribution of MS prevalence and its relationship with spatial patterns of air pollution. By collecting records of 2,188 patients who met the criteria for a definite diagnosis of MS from 2003 to 2013, we used a regression model to estimate long-term exposure to NO_x_ in MS patients. The results revealed a cluster of MS epidemic cases in Tehran, and the spatial distribution of NO_x_ exposure was significantly different from that of MS epidemic cases (*P* < 0.001), demonstrating the potential role of long-term exposure to NO_x_ as an environmental risk factor for MS (107).

### The mechanisms by which air pollution induces multiple sclerosis

Although the influence of air pollution on the pathogenesis of MS is not completely clear, exposure to polluted air can stimulate a variety of mechanisms, which may enhance the incidence and severity of MS. The main underlying mechanism is oxidative stress following the inflammatory reaction of the immune disorder, which leads to neuroinflammation and destroys the normal balance between immunity and self-tolerance (109). After air pollutants are inhaled and deposited in the human respiratory tract, they induce and maintain chemical reactions, producing ROS and nitrogen reactive species (RNSs). The imbalance between pro-oxidant species (ROS/RNS) and antioxidant species leads to the production of pro-inflammatory cytokines by alveolar macrophages and airway epithelial cells. These cytokines can recruit and activate neutrophils, monocytes, and dendritic cells and stimulate adaptive immune responses, such as Th1 and Th17 inflammatory responses (110). After these events, uncontrolled inflammatory reactions lead to cell death and self-antigen release, which stimulates the production of self-attacking T cells by enhancing antigen presentation and promotes cell entry into the central nervous system (102, 111). Therefore, OS induces systemic inflammation and immune imbalance through these pathways, representing as a risk factor for MS.

**Figure 2:**
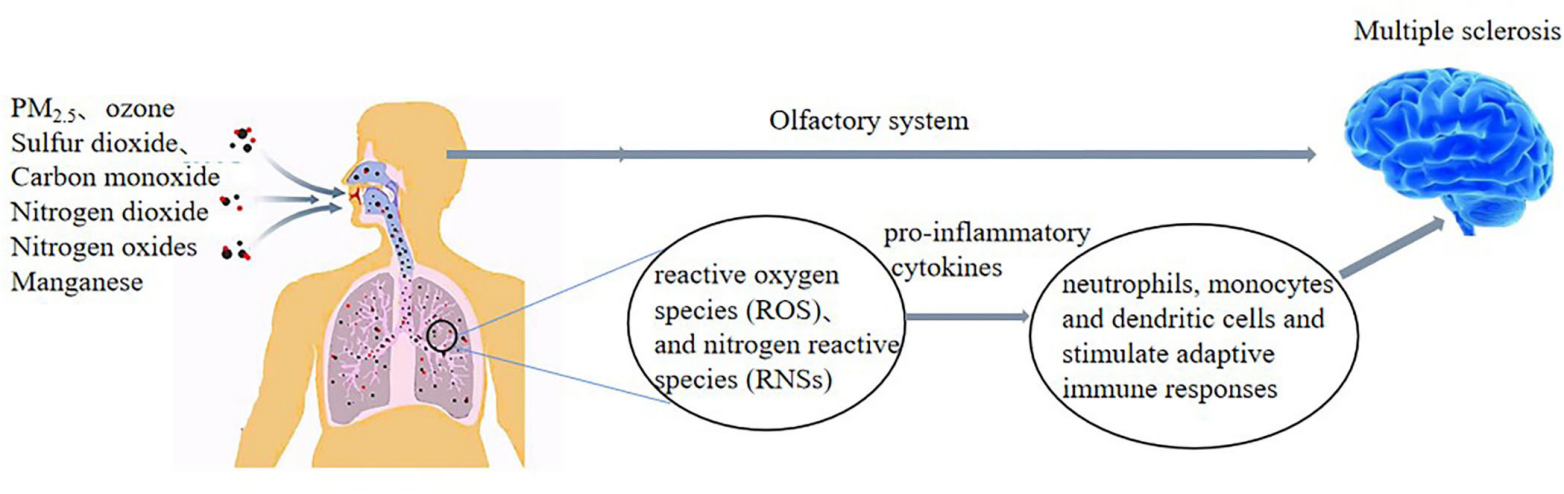
Air pollution may trigger multiple sclerosis although an inflammatory reaction. In addition, air pollutant transfer through the olfactory system and passage through the blood–brain barrier is another possible mechanism that aggravates multiple sclerosis.

In addition, the transfer of air pollutants through the olfactory system and their ability to cross the blood–brain barrier is another possible mechanism that exacerbates MS disease. Using this route, air pollutants can directly reach the brain parenchyma, and after causing epigenetic changes in glial cells, induce the production of pro-inflammatory cytokines from innate and adaptive immune cells and autoimmunity in the cerebrum (102, 108, 110, 112) (see Figure 2).

### Air pollutants and epilepsy

Epilepsy is a common neurological disorder characterized by abnormal neuronal excitability and unpredictable recurrent seizures (113, 114). Epilepsy is a wide-ranging and complex disease affecting more than 70 million people, ranging from newborns to the elderly (115). Research on the health effects of air pollutants on epilepsy is limited. Based on a single-center study in China, Xu et al. found that SO_2_ was associated with an increase in outpatient visits to epilepsy clinics with an increase in 3.55% (95% CI: 1.93–5.18%) for every 10 μg/m^3^ increase in SO_2_ (116). A nationwide study conducted by Cakmak et al. in Chile from 2001 to 2005 found that SO_2_ was associated with an increase in the number of epilepsy hospitalizations (117). Only a few articles have reported the impact of SO_2_ on epilepsy. It is urgent to further study the impact of air pollution on the brain to better estimate the burden of epilepsy caused by SO_2_.

### Heavy metal damage and neurological disorders

#### Arsenic and neurological disorders

With the rapid development of industrialization, mining, the smelting of non-ferrous metal mines, and the mass production and use of coal, pesticides, pigments, and dyes, the problem of arsenic pollution in the air has become increasingly serious, representing a global public health problem (118). Arsenic in the air can enter lung tissue through respiration and cause acute or chronic arsenic poisoning in humans. Long-term exposure to arsenic may lead to chronic arsenic poisoning, resulting in pathological changes in various tissues and organs of the human body (119). Arsenic poisoning is also closely related to damage to the nervous system. Arsenic poisoning can seriously affect the differentiation function of adult neural stem cells, impair neurogenesis, and eventually lead to poor learning and memory. Hong et al. cultured pluripotent P19 cells *in vitro* and found that arsenic poisoning can significantly reduce the ability of P19 cells to differentiate into neurons by inhibiting the WNT/β-catenin signaling pathway (120). Arsenic poisoning also increased apoptosis in cultured human neural stem cells and inhibited their ability to differentiate into neurons (121). In an intervention experiment on newborn rats, arsenic poisoning led to impaired differentiation of immature neurons in the outer layers of granulosa cells and pyramidal cells, resulting in neurogenesis abnormalities and increased anxiety-like behavior in rats (122). Liu et al. also found that arsenic poisoning significantly reduced cell proliferation and maturation in the SGZ region of mice. Arsenic poisoning resulted in the proliferation of hippocampal neurons in adult rats and inhibited adult neurogenesis (123).

## Conclusion

With the acceleration of modern industrialization and urbanization, the levels of various forms of pollutants, such as atmospheric particulate matter, O_3_, sulfur oxides, carbon oxides, NO_x_, and heavy metals, have increased dramatically. Based on this review, we can conclude that air pollutants can damage the nervous system directly or indirectly. For example, increased PM_2.5_ concentrations can increase the incidence of CVD, AD, MS, and PD, and increased O_3_ concentrations can increase the incidence of AD, MS, and PD. Regarding the mechanism of air pollution in central nervous system disorders, some studies demonstrate an association between neuroinflammation and long-term exposure to air pollution. Inflammation is a major contributor to many neurodegenerative diseases, and pollutants may actually induce inflammation.

However, at present, there is still no clear and unified statement on the epidemiological and pathological effects on the components of air pollution and central nervous system injury, and further research is needed given that this information is crucial for human health. It also helps the government develop policies for the early diagnosis and long-term follow-up of neurological disorders for people living in contaminated areas.

## Author contributions

CF, HZ, DK, JR, and JC designed the study and were the main drafters of the manuscript. All authors participated in the analysis and discussion under the leadership and instruction of JC. All authors read and approved the final manuscript.

## Funding

This study was funded by the National Natural Science Foundation of China (81502899 and 82103879), Guangdong Basic and Applied Basic Research Foundation (2021B1515140032), Discipline Construction Project of Guangdong Medical University, Discipline Construction Project of Guangdong Medical University (4SG22021G), the Fund for Ph.D., Researchers of Dongguan People's Hospital (K202001), and Science Foundation of Traditional Chinese (20222104).

## Conflict of interest

The authors declare that the research was conducted in the absence of any commercial or financial relationships that could be construed as a potential conflict of interest.

## Publisher's note

All claims expressed in this article are solely those of the authors and do not necessarily represent those of their affiliated organizations, or those of the publisher, the editors and the reviewers. Any product that may be evaluated in this article, or claim that may be made by its manufacturer, is not guaranteed or endorsed by the publisher.
